# Promoting Complex Problem Solving by Introducing Schema-Governed Categories of Key Causal Models

**DOI:** 10.3390/bs13090701

**Published:** 2023-08-23

**Authors:** Franziska Kessler, Antje Proske, Leon Urbas, Micah Goldwater, Florian Krieger, Samuel Greiff, Susanne Narciss

**Affiliations:** 1 Faculty of Psychology, Technische Universität Dresden, 01069 Dresden, Germany; antje.proske@tu-dresden.de (A.P.); susanne.narciss@tu-dresden.de (S.N.); 2 Department of Electrical Engineering, Technische Universität Dresden, 01069 Dresden, Germany; leon.urbas@tu-dresden.de; 3 School of Psychology, University of Sydney, Camperdown, NSW 2050, Australia; micah.goldwater@sydney.edu.au; 4 Faculty of Rehabilitation Sciences, TU Dortmund University, 44227 Dortmund, Germany; florian.krieger@tu-dortmund.de; 5 Department of Behavioral and Cognitive Sciences, University of Luxembourg, 4366 Luxembourg, Luxembourg; samuel.greiff@uni.lu; 6 Center of Tactile Internet with Human in the Loop (CeTI), Technische Universität Dresden, 01069 Dresden, Germany

**Keywords:** relational reasoning, transfer, complex problem solving, schema-governed category

## Abstract

The ability to recognize key causal models across situations is associated with expertise. The acquisition of schema-governed category knowledge of key causal models may underlie this ability. In an experimental study (*n* = 183), we investigated the effects of promoting the construction of schema-governed categories and how an enhanced ability to recognize the key causal models relates to performance in complex problem-solving tasks that are based on the key causal models. In a 2 × 2 design, we tested the effects of an adapted version of an intervention designed to build abstract mental representations of the key causal models and a tutorial designed to convey conceptual understanding of the key causal models and procedural knowledge. Participants who were enabled to recognize the underlying key causal models across situations as a result of the intervention and the tutorial (i.e., causal sorters) outperformed non-causal sorters in the subsequent complex problem-solving task. Causal sorters outperformed the control group, except for the subtask *knowledge application* in the experimental group that did not receive the tutorial and, hence, did not have the opportunity to elaborate their conceptual understanding of the key causal models. The findings highlight that being able to categorize novel situations according to their underlying key causal model alone is insufficient for enhancing the transfer of the according concept. Instead, for successful application, conceptual and procedural knowledge also seem to be necessary. By using a complex problem-solving task as the dependent variable for transfer, we extended the scope of the results to dynamic tasks that reflect some of the typical challenges of the 21st century.

## 1. Introduction

The advancement of new digital transformation technologies is profoundly changing the workplaces of the 21st century [1]. We are observing a massive increase in automation as well as an increase in the flexibility of novel systems (e.g., in the chemical process industry [2]). As a consequence, an increasing number of job responsibilities have moved from well-defined routine tasks such as supervisory control of the system [3] to those of a more dispositive nature that require active problem solving [4,5,6]. But work is by far not the only area of life that is becoming more volatile and complex; everyday life is shaped by rapidly evolving technical devices and technology, accelerated communication, and the possibility to access comprehensive information from the internet at almost any time via mobile devices [7]. These developments demand a lot from us [8]. In order to deal with such rapid changes in a complex world, individuals must be adept at transferring knowledge from the past to the present and adjusting their solution approach effectively. For example, when confronted with a task or a problem (i.e., target) and not knowing how to solve it, a good strategy would be to search memories for past problems based on the same underlying causal relationship or principle (i.e., source) in order to derive a suitable solution strategy [9]. The source can be mapped to the target by structurally matching the corresponding elements [10,11], thus allowing the individual to draw meaningful conclusions about the behavior and the solution of the target by analogical reasoning. Accordingly, knowledge transfer can contribute to more efficient problem solving by obviating the necessity to devise a new solution strategy from scratch [9].

Knowledge can be transferred across domains, physical settings, or modalities [12]. Given the utility of transferring knowledge in our quickly changing world, it has been suggested to be core of the “21st century skills”, which are a set of particularly relevant skills in today’s world [13,14]. However, transfer is challenging. People oftentimes struggle to recognize the relevance of previously learned materials when the problem at hand differs in objects, settings, or context [15]. According to Novick [9], there are several stages in the process of analogical transfer that potentially pose an obstacle and therefore make it difficult. First, people may fail to recognize the relevance of a previous situation or problem for the target and their memory search is unproductive. Second, they become distracted by a case from the past with matching superficial similarity, which does not follow the same principle as the target and, hence, is irrelevant for reasoning about the current problem or may even be misleading. Third, the user does not know how to apply the retrieved case to the target.

A large body of research has shown that people rarely experience spontaneous reminding based on a deep causal structure or underlying principle, even when it would be helpful for solving the problem at hand [16,17]. Instead, people are more likely to be reminded of past experiences similar in surface features such as objects, locations, or characters [18,19,20]. There is a discrepancy between what people consider to be a sound inferential match and what they are likely to retrieve from memory. Gentner et al. (1993) demonstrated that people tend to judge structurally equivalent cases as “more sound” matches, as predicted by structure mapping theory [10]. But when it comes to retrieval from long-term memory, it is mainly driven by surface similarity and is weakly influenced by structural similarity [20]. A proposed reason for this misbalance between the actual retrieved cases (surface matches) and the cases one desires in terms of usefulness for solution (relational match) is that relational retrieval is much more computationally demanding than retrieval driven by matching superficial features [21,22]. Consequently, people oftentimes resort to the easy and quick search of memory by matching superficial features even though the results are more likely to be misleading.

Increasing experience and understanding in a domain substantially alters the way new problem situations are perceived, represented, and categorized [23]. In this work, we set out to study a specific form of portable knowledge that is associated with expertise: the formation of abstract mental representations of key causal models. It has been argued that this is the reason for experts’ improved ability to recognize important causal models in a variety of situations across different domains [24,25,26]. We aim to shed light on how the ability to recognize key causal structures across situations relates to performance in solving complex problems, study the effectiveness of learning tasks that are designed to target and foster this ability, and evaluate its effects on performance in a complex problem-solving task. For this, we build on results from a previous study from our lab [27]. In the following paragraphs, we briefly discuss the relevance of relational reasoning, followed by an overview of the results from expertise research highlighting the role of knowledge organization in terms of underlying relational content rather than superficial features and how it pertains to spontaneous transfer, discuss approaches to promote the acquisition of such expert-like knowledge organization, and, based on these considerations, delineate the reasoning for our study.

## 2. Teaching Relational Reasoning

Relational reasoning is defined as “the ability to discern meaningful patterns within informational streams” [28]. It enables the extraction of regularities in terms of underlying relational structure among situations that bear little superficial resemblance and therefore constitutes the basis for successful knowledge transfer [29]. Individuals’ capacity to reason relationally is linked to cognitive capacity measures such as fluid intelligence, working memory, or executive functioning [29]. Scores in the standard measure of fluid intelligence predict spontaneous analogical transfer [30] and relational mapping [31]. The importance of cognitive capacity has been stressed by a number of computational models of analogical reasoning [22,32,33]. At the same time, there is evidence that relational reasoning is trainable with the right set of learning experiences [28,34]. Kubicht et al. [30] showed that the relationship between fluid intelligence and analogical reasoning is moderated by the provision of learning materials designed to foster an understanding of the source. An inductive reasoning training program aimed at teaching to infer and generalize underlying relational patterns enhanced the ability to transfer the relational content and improved fluid intelligence at a six months delayed post-test [35,36,37]. Inductive reasoning was defined as a cognitive process that “enables one to detect regularities, rules, or generalizations and, conversely, to detect irregularities” ([35] p. 1), and therefore has a large overlap with the conception of relational reasoning. Hence, there is reason to believe that the ability to reason relationally is not purely dependent on inter-individual differences in cognitive capacity, but rather can be fostered with the right learning tasks and cognitive activities.

## 3. Differences between Novices and Experts’ Knowledge Organization

Card sorting tasks that ask participants to cluster descriptions of phenomena in terms of their similarity are frequently used for the assessment of knowledge organization [38]. In an influential study, Chi et al. [24] showed that with increasing expertise in physics, participants were more likely to cluster a series of physics problems according to the underlying principle (e.g., conservation of energy), while novices tended to cluster according to superficial feature-based characteristics (e.g., problems containing specific features such as springs or pulleys). Similar results have been found for the domain-specific conceptual expertise in chemistry [39,40,41] and biology [42,43]. The findings of these card sorting paradigms all underpin a gradual change in conceptual organization as learners acquire more domain knowledge, which is referred to as relational shift [40,44,45].

One finding that demonstrates the generalization of domain-specific conceptual expertise across domains was reported by Rottman et al. [26]. They designed the Ambiguous Card Sorting Task (ACST) that requires participants to cluster different descriptions of real-world phenomena. The descriptions vary along two dimensions: (a) the underlying causal model (e.g., common cause or causal chain) and (b) the scientific content domain (e.g., biology or economics). Hence, the sorting for each description can be performed either according to the content domain or according to underlying causal model of the phenomenon [26]. They showed that advanced students from an integrated science program (i.e., physical science experts) were more likely to spontaneously categorize phenomena according to their underlying causal model. Intriguingly, they were not only more likely to detect the underlying causal models in descriptions of their field of expertise, but also in other domains such as economics, indicating a generalization of the key causal models. The authors argued that physical science experts had acquired abstract mental representations (i.e., abstract schemata). By habitually interpreting novel situations in terms of these abstract schemata, experts are more likely to recognize novel situations outside of their domain of expertise as an instance of these abstract schemata [16]. Further, it accounts for experts’ higher likelihood to spontaneously retrieve relational matches from memory and subsequent facilitated cross-domain transfer [9]. These results all suggest that prior relational knowledge has a top-down influence on the way a novel situation representation is construed [46,47].

The differential sorting strategy in the card sorting tasks reflects qualitative differences in the categories that novices and experts impose on situations [48]. Experts use relational categories whose membership is defined by the relational content of their exemplars [49]. In contrast, novices apply feature-based categories that are defined by the internal features of the entities [50,51]. Consequently, members of the same feature-based category have high perceivable similarity [52]. The membership of relational categories is not determined by superficial similarity, but relational content, and therefore exemplars likely bear low superficial resemblance to each other [52]. Relational categories are differentiated into role-governed and schema-governed categories [52]. Members of role-governed categories all occupy the same role within a relational structure, while members of schema-governed categories are all based on the same relational system or principle [53]. Consider the relationship “x sells something to y”. The relationship “selling something” defines the roles of x and y within the relational system “sale”: x becomes a vendor and y becomes a customer, both examples of role-governed categories. The variables x and y act as placeholders for all sorts of things, people, or objects that could all be instantiations of the according role-governed category. The overall process of “sale” refers to the relational system or schema as a whole and henceforth represents a schema-governed category [52].

## 4. Learning Relational Categories

Gaining understanding and expertise changes the way new problem situations are perceived, represented, and categorized [23]. Categorization is critical, as it determines what schemata and procedures are subsequently activated [23]. That is why categorization according to underlying principle, typical in experts, is likely to activate conceptual knowledge and procedures on how to solve such situations, whereas categorization in terms of superficial features is likely to activate knowledge of situations that cannot be solved by the same procedure, which can, in the worst case, be misleading [9].

The bottleneck for successful problem solving is to recognize which prior knowledge can be applied to the current problem at hand. The building of relational categories around abstract schemata can help overcome this difficulty by supporting useful problem classification, thus mitigating some of the dependence on superficial features in analogical retrieval [54]. Therefore, devising learning tasks that support the reorganization of knowledge in terms of relational categories may constitute a promising way to facilitate and promote knowledge transfer [54]. In this context, Kurtz and Honke [55] proposed the idea of category status that posits “to the extent that the form of a knowledge representation is more [relational] category-like, the knowledge will be easier to access under the critical conditions of high structural match and low superficial match” [55]. In fact, they showed that participants who performed a card sorting task with the goal of building up a relational category around a principle elicited more spontaneous transfer in a subsequent transfer task based on the introduced principle compared to a group that completed an analogical comparison task to exemplify the principle. This is particularly striking, as analogical comparison is considered the gold standard in enhancing schema abstraction and, thus, transfer [56]. The authors argued that the process of categorization is effective beyond schema abstraction because it not only makes the representation more abstract, but also encompasses a reorganization of knowledge that creates a pathway for accessing relevant knowledge, e.g., analogous cases by categorizing a situation as belonging to a particular type or class (i.e., category). The recognition of a situation as a member of a relational category can act as a pointer to or internally generated hint for the relevant relational memory content. In a similar vein, Jamrozik and Gentner [57] demonstrated that introducing labels for causal model categories (they call them relational schema labels) facilitates relational retrieval. They claim that providing labels for the relational schemata can make memory search more efficient, hence facilitating relational retrieval and subsequent transfer.

Using these insights helps identify targets for the design of effective learning tasks, which is one of the central goals of this study. Earlier accounts have demonstrated that the ability to categorize situations according to their underlying principle or causal relationship can be trained and is associated with better problem-solving performance, e.g., for statistical problems [58] and for physics problems [59,60]. Goldwater and Gentner [25] investigated learning tasks that promote an expert-like ability to organize situation descriptions according to their underlying causal structure. In their study, they concentrated on four very basic, but ubiquitous key causal models: common cause, common effect, causal chain, and positive feedback system. They found the combination of direct explication of the key causal models followed by a guided structural alignment task to be most effective in increasing the ability to recognize key causal models across different situation descriptions in the Ambiguous Card Sorting Task (ACST) [26]. The authors attributed this expert-like relational shift to the fact that participants learned to use the schema-governed categories of the key causal models to make sense of novel situations.

## 5. Effects of Learning Schema-Governed Categories on Complex Problem Solving

Recently, the practical educational relevance of the findings by Goldwater and Gentner [25] has been investigated [27]. In particular, the question of whether enhancing the ability to recognize key causal models across situations would also translate into better performance in problem solving for tasks that are based on these key causal models was addressed. Complex problem-solving tasks are designed to tap into real-world problem solving when dealing with unfamiliar situations with no obvious solution strategy available, or when the information required for a solution is unavailable [6]. Complex problem-solving tasks impose specific requirements on the problem solver, such as anticipation of the evolution of the system state and the planning of individual sub-steps necessary to reach a goal state [61,62]. In the first phase, participants have to actively explore the problem situation in order to gain knowledge of the underlying causal relations of the situation (i.e., *knowledge acquisition*) [63]. This is followed by the phase of controlling the system by the manipulation of its constituent elements (i.e., *knowledge application*) [63]. These two facets, *knowledge acquisition* and *knowledge application*, have been shown to be empirically separable, yet highly correlated [64]. Complex problem-solving tasks are usually implemented as computer-based simulations (called microworlds) designed to mirror the essential characteristics of real world problems [65]. Such microworlds are a means of bridging the gap between field and laboratory research [66]. Therefore, using complex problem-solving tasks enabled the expansion of the scope of the results to dynamic, non-routine situations typical for the 21st century, rather than studying the effects on static problems.

Kessler et al. [27] used the MicroDYN approach [67] as a measure for complex problem solving. They found a significant positive correlation between the ability to recognize key causal models and performance in the complex problem-solving tasks. While they did not find a direct effect of the intervention on performance, they found an indirect-only effect of the intervention via the ability to recognize the key causal models on performance. Participants who shifted their sorting behavior to be more expert-like, i.e., who sorted cards in the ACST mainly according to the underlying causal model (i.e., causal sorters), outperformed non-causal sorters across all groups in the complex problem-solving task. In a follow-up analysis not reported in the paper, they found that the causal sorters in the intervention group performed better than the non-causal sorters in the *knowledge acquisition*, but not the *knowledge application* phase. This differential effect is in accordance with theoretical considerations. During the *knowledge acquisition* phase, the ability to detect key causal models across situations can facilitate the recognition of the according key causal models in the microworlds. However, during the *knowledge application* phase, the correct causal model is provided to the participants in MicroDYN and the task is to control the microworld through the manipulation of the input variables. In that case, recognition of the underlying causal model will no longer be sufficient; instead, a deep conceptual understanding of the behavior and implications of the key causal models is required for effective system control. Interestingly, the participants in the control group (CG) who engaged in a predominantly causal sorting strategy without receiving the intervention were among the high performers in *knowledge acquisition* as well as *knowledge application*, suggesting that those participants entered the experiments as “experts” who were not only adept at recognizing the causal models, but also possessed a sufficient understanding of the behavior of the models to successfully control the system. However, since only four participants fell into this subgroup (11% of participants in the CG), the results should be interpreted with caution.

## 6. Current Study

The pattern of results led to the conjecture that the intervention alone is insufficient to induce expert-like performance. The ability to detect the underlying key causal model can only exert its full beneficial effect if associated conceptual knowledge and procedures are available in the schemata to be invoked by the according categorization. To test this notion, we designed a tutorial targeted to promote a deep conceptual understanding of the behavior of each of the key causal models. Specifically, the tutorial is supposed to advance the understanding on the dynamic behavior of the key causal models that form the basis for successful mental simulation, prediction of future behavior, and derivation of suitable strategies for system control [68]. The idea is that the combination of intervention (i.e., construal of schema-governed categories) and tutorial (i.e., elaboration of conceptual knowledge and acquisition of procedural knowledge for system control) will foster the performance in the *knowledge acquisition* and *knowledge application* phase. This is because the learning tasks foster the acquisition of expert-like knowledge organization in terms of schema-governed categories and associated conceptual and procedural knowledge, thus allowing for the recognition of the underlying causal model and instantiating its principles to solve a given situation [60]. We employed a 2 × 2 experimental design with the factors of intervention (yes/no) and tutorial (yes/no) in order to test the effects of each of the factors and their interaction on the ability to detect the key causal models in the ACST as well as on the performance in the complex problem-solving subtasks *knowledge acquisition* and *knowledge application*.

The study design of our last study did not allow for the differentiation between the participants who entered the experiments with a spontaneous expert-like (i.e., high) ability to detect the key causal models and those who entered as non-causal sorters and then shifted their sorting pattern as a result of the intervention. However, it is likely that the effects on performance are more pronounced for participants who brought a high ability to recognize key causal models to the experiment than for those who just acquired that ability through the intervention and/or tutorial. In order to forestall the confounding influence of different ability levels upon the experiment start, we designed a parallel version of the adapted ACST [26] that we used in the last study. For this, we designed 16 phenomena descriptions that systematically varied in content domain and underlying causal model. Two parallel ACSTs enable two consecutive assessments of the ability to detect the key causal models: one at the beginning of the experiment and one after the intervention and/or tutorial, thus allowing the identification of participants who entered the experiment as causal sorters (i.e., initial causal sorters) and pooling them into a separate experimental group. They served as an additional control group for the special case of initial causal sorters (i.e., experts). By removing them from the treatment groups, it is possible to study the impact of a newly acquired ability to recognize important causal models elicited by the intervention and tutorial on complex problem-solving performance.

### Research Question and Hypotheses

The current study seeks to further the findings of our previous study on the relationship between the learning schema-governed category knowledge of key causal models and performance in complex problem-solving tasks based on the key causal models. The implementation of the initial ACST allows the identification of those participants with a high spontaneous ability to categorize most of the situation descriptions according to the underlying causal model, i.e., initial causal sorters. Based on the core idea of the experiment, we conjecture that initial causal sorters can be considered “experts” who habitually interpret new situations in terms of the schema-governed categories of the key causal models. We assume that expertise entails the availability of conceptual knowledge that can be activated as a result of schema–governed categorization. We predict that this expert-like knowledge representation and organization in terms of schema-governed categories is beneficial for complex problem-solving performance. Initial causal sorters will be identified and pooled together as an expert baseline group. Participants in that group will not receive any further treatments. We predict that initial causal sorters are among the high performers in the overall sample.

**H1.**  *Initial causal sorters are among the high performers in the sample for knowledge acquisition and knowledge application.*

In a next step, we want to explore whether it is possible to induce such an expertise-like knowledge representation and organization using learning tasks that (a) have been shown to enhance the learning of schema-governed categories of the key causal models (i.e., the structural alignment and explication intervention) and (b) foster a conceptual understanding of the key causal models (i.e., the tutorial). The second ACST tests the increase in causal sorts as a result of the treatment tasks. This extends the findings of previous studies using the intervention [25,27] by identifying the direct increase as a result of the intervention, without the confounding influence of initially high levels of causal sorting. We assume that the tutorial will also promote schema-governed category learning and therefore enhances the amount of causal sorting. We predict that the group that receives both intervention and tutorial will show the largest increase in causal sorting because the intervention and tutorial are designed to provide an ideal basis for the learning of schema-governed categories of the key causal models as well as enhancing conceptual understanding.

**H2a.**  *The ability to recognize the key causal models (i.e., number of causally sorted ACST cards) increases after intervention and tutorial.*

**H2b.**  *The highest increase in causal sorting is in the group that received the structural alignment and explication intervention as well as the tutorial.*

Concerning the effect of the intervention and the tutorial on performance in the complex problem-solving tasks, we hypothesize that beneficial effects will become primarily apparent for those participants who adopt a predominantly causal sorting pattern (i.e., causal sorters) as a result of the treatments. We predict that the causal sorters in the experimental groups will show better performance for *knowledge acquisition* than non-causal sorters, because they are adept at recognizing the underlying key causal models across situations. For the performance indicator *knowledge application*, we expect more differentiated results: causal sorters who underwent the tutorial should outperform non-causal sorters, because they were given the chance to enhance their conceptual understanding of the key causal models and procedural knowledge on how to control systems based on the according key causal models. In contrast, the causal sorters who only received the intervention should be enabled to recognize the causal models, but were not equipped with further conceptual and procedural knowledge and thus should not perform better than the non-causal sorters in the *knowledge application* phase (i.e., replication of results from previous study).

**H3a.**  *Participants who shift their sorting strategy to be mainly causal (i.e., causal sorters) after receiving the intervention (Int), the tutorial (Tut), or both (Int and Tut) outperform participants who do not show this shift in sorting (i.e., non-causal sorters) in the knowledge acquisition phase.*

**H3b.**  *Participants who shift their sorting strategy to be mainly causal (i.e., causal sorters) after receiving the tutorial (Tut; Int and Tut) outperform participants who do not show this shift in sorting (i.e., non-causal sorters) in the knowledge application phase. There are no differences in knowledge application between the causal sorters in the group that received only the intervention (Int) and non-causal sorters.*

In order to further corroborate the impact of adopting a mainly causal sorting strategy, we compare the causal sorters in the treatment groups (i.e., participants who shifted as a result of intervention and/or tutorial) to the two baseline conditions: the CG and the group of initial causal sorters. Compared to the CG, we expect causal sorters in the treatment groups to show better performance in the *knowledge acquisition* phase. For the *knowledge application* phase, we expect only the causal sorters from the groups that received the tutorial (Int and Tut; Tut) to perform better than the CG.

**H4a.**  *The knowledge acquisition of causal sorters from the treatment groups is better than for the baseline condition (CG).*

**H4b.**  *The knowledge application of causal sorters in the two groups that received the tutorial (Int and Tut; Tut) is better than for the CG.*

**H4c.**  *The knowledge application of causal sorters in the intervention group (Int) does not differ from the CG.*

Likewise, we assume that causal sorters in the treatment groups should show comparably high *knowledge acquisition* as initial causal sorters. For *knowledge application*, we expect causal sorters who received the tutorial (Int and Tut; Tut) to show comparably high performance as the initial causal sorters, while causal sorters who only received the intervention are expected to perform worse than initial causal sorters in *knowledge application*.

**H5a.**  *The knowledge acquisition of causal sorters does not differ in the experimental groups compared to initial causal sorters.*

**H5b.**  *The knowledge application of causal sorters in the two groups that received the tutorial (Int and Tut; Tut) does not differ from initial causal sorters.*

**H5c.**  *The knowledge application of causal sorters in the intervention group (Int) is worse than the performance of initial causal sorters.*

We also predict that participants benefit more from the tutorial if they have already built up abstract schemata of the key causal models. This is because it would allow the assimilation of additional conceptual and procedural knowledge into existent schemata [69]. The final task of the tutorial was a card sorting task in which cards with certain characteristics of causal behavior had to be assigned to their corresponding causal models. The performance in this task can serve as an indicator for the success of the tutorial as it captures whether participants have successfully linked the conceptual knowledge to the according key causal model schemata. We therefore hypothesize that the performance in the card sorting task at the end of the tutorial is higher for the group that underwent the intervention prior to the tutorial, i.e., group Int and Tut.

**H6.**  *The score in the card sorting tutorial task is higher in group Int and Tut compared to group Tut.*

## 7. Methods

### 7.1. Participants

We recruited participants using the online platform Prolific (www.prolific.co) and via advertising on the psychology experiment pool of the TU Dresden. We aimed to keep the number of participants from the two sources equal for each of the experimental conditions. To participate in the experiment, the participants had to be aged between 18 and 35 years old, their native language had to be German, they had to have a higher educational qualification of Abitur (or equivalent) or above, and they were not allowed to have previously participated in experiments conducted by our laboratory using the materials used in this study. We recorded 229 complete datasets on Labvanced; 35 participants failed to complete the following MicroDYN task (due to temporary problems with the hosting server, or to technical problems with the task). In total, 194 participants submitted a complete dataset. Thereof, six were excluded because they failed to meet the abovementioned inclusion criteria (e.g., not meeting the requirement of a higher educational qualification of Abitur or above, indication that German is not their mother tongue, older than 35 years). We also excluded five datasets in which the participants did not complete the intervention task according to the instructions, i.e., failed to complete all four structural alignment tasks included in the intervention. The completion of these four tasks was essential for the formation of abstract schemata as intended by the intervention. In total, 183 datasets were included in the following data analyses (female = 137, diverse = 3, male = 43; mean age = 22.4, SD = 3.31; for 28 participants the age was not recorded due to technical issues).

### 7.2. Design and Procedure

**Figure 1:**
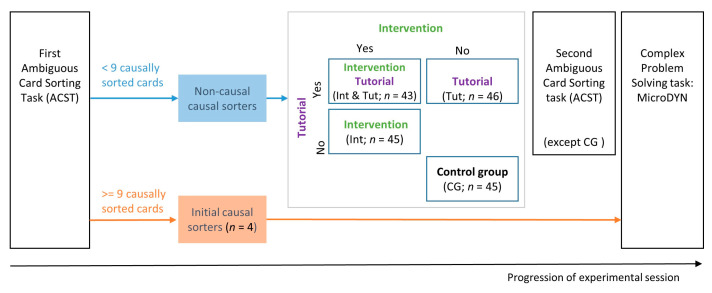
Experimental design and experimental procedure of the study. The diagram shows the sequence of tasks and treatments that the respective experimental groups underwent during the experimental session.

We employed a 2 × 2 between-subjects experimental design with the two factors of intervention (yes/no) and tutorial (yes/no). This resulted in four experimental groups: one group that received the intervention (Int; *n* = 45), one that received the tutorial (Tut; *n* = 46), one that received both intervention and tutorial (Int and Tut; *n* = 43), and one control group that did not receive either (CG; *n* = 45). The experimental design also included a fifth group serving as a special case of control group: participants who sorted nine or more cards according to an underlying causal model in the first ACST were pooled together to the additional group of initial causal sorters (ICS; *n* = 4; see Figure 1).

The experimental procedure for each respective experimental group was as follows (see Figure 1): all participants started with the initial ACST. The participants who sorted nine or more cards using a causal strategy were classified as initial causal sorters and assigned to a separate group, i.e., initial causal sorters (ICS). They did not undergo any further experimental interventions and continued directly with the complex problem-solving task. In the intervention group (Int), the participants proceeded with the intervention, followed by the second ACST and the complex problem-solving task. The group that received the intervention and tutorial (Int and Tut) proceeded with the intervention, followed by the tutorial, second ACST, and the complex problem-solving task. The tutorial-only group (Tut) progressed with the tutorial, and then performed the second ACST and finished with the complex problem-solving task. The control group (CG) continued with the complex problem-solving task immediately after the first ACST. We did not include a second ACST in the control group in order to minimize the impact that the ACST itself may have on the construal of schema-governed categories [55]. The order of the versions (ACST and ACST_p) was balanced between the participants within each experimental group.

The experiment was partly implemented on the online experiment platform Labvanced [70]. The participants accessed the online experiment via a link. Prior to starting the experimental tasks, the participants were required to confirm they met the inclusion criteria. The participants received the study information as a PDF file and were informed about data security measures. Only participants who provided consent by ticking a box could proceed with the experiment. At the end of the experimental part implemented in Labvanced, the participants were provided with a link that redirected them to the complex problem-solving tasks. In order to account for the different durations of the experimental conditions, we created individual studies on Labvanced for each condition. The participants could decide on the duration of the experimental session and were assigned to an according condition. The information provided to the participants was identical for each condition in order to minimize self-selection processes. Participation in the study was compensated with EUR 10, 15, or 20 or 1 h, 1.5 h, or 2 h class credit depending on the duration of the experimental condition. The experimental protocol was approved by the local Ethics Committee (SR-EK-280052021). Statistical analyses were conducted using JASP version 0.16.1 [71] and MATLAB [72].

### 7.3. Material

All materials in this experiment needed to be based on the same key causal models. The intervention by Goldwater and Gentner [25] addressed the key causal models’ common cause, common effect, causal chain, and positive feedback system. However, the MicroDYN tasks that we used as a measure for complex problem solving did not allow the implementation of positive feedback systems. Instead, we implemented the conceptually very similar causal structure of self-enhancing systems. In order to align all materials in this study, we rephrased the positive feedback system to a self-enhancing system in the intervention and ACSTs.

**Ambiguous card sorting task (ACST)**: We used an adapted version of the original ACST by Rottman et al. [26] as a measure of the ability to detect key causal models across situations from different fields. The participants were provided with 16 cards with different situation descriptions. (In the original version by Rottman et al. [26], 25 cards were included. However, the descriptions of the electrical engineering domain were used in the intervention and the descriptions with underlying negative feedback systems were not included in the intervention; we only used the remaining 16 cards, as in Goldwater and Gentner [25]). Each description differed along two dimensions: content domain and underlying key causal model. Four cards were shown to the participants as the headers of columns plus one additional header with the label “other”. The participants were instructed to read the cards, and then to sort each of the remaining 12 cards to one column “based on how well the description on the card goes with the initial card’’ [26]. In case they thought the card did not match any of the header cards, they were instructed to place the card in the “other“ column. As each situation description differs in content domain as well as the underlying causal model, the sorting strategy can either be based on matching the underlying key causal model (i.e., causal sorting) or content domain (i.e., domain sorting). The 12 cards were provided in the same semi-randomized order to each participant.

The number of cards sorted according to the underlying causal model served as a measure for the ability to detect the key causal models across situations. Previous studies that used card sorting tasks to assess differences between experts and novices reported a range of 60–95% causal sorts in experts [24,26,39,42,73]. For example, in Chi et al. [24] (study two) the physics professors sorted 19/20 physics problems (95%) according to the underlying principle, the graduate student 17/20 problems (85%), an intermediate novice 13/20 problems (65%), and a novice 0/20 problems (0%) according to the underlying physics principle. As we were particularly interested in the effects of adopting an expert-like causal sorting strategy, we classified the participants as causal sorters if they showed a profound relational shift. We decided to use 75% causal sorting (nine or more cards out of twelve) as a cut-off for classification as a causal sorter, as this implies that participants identified three out of four key causal models reliably or all four key causal models at least to a fair extent.

**Parallel ACST** (ACST_p): We constructed 16 situation descriptions. Each description differed in content domain (economics, biology, environmental science, and engineering/tech) and in underlying causal model (common cause, common effect, chain, self-enhancing system) and, hence, allowed for a domain or causal sorting strategy of each of the descriptions. Please note that two of the situation descriptions were taken from the original ACST by Rottman et al. [26] that we did not use in the adapted version in our previous experiment [27]. In a pre-study (*n* = 62), we showed that the original and the parallel version did not differ in the number of cards sorted by domain or causal model, and henceforth considered the two versions parallel.

**Intervention**: We used an adapted version of the explication and structural alignment intervention by Goldwater and Gentner [25]. We rephrased the positive feedback system to a self-enhancing system. The intervention comprised two steps: the explication of the key causal models and a guided structural alignment task. The goal of the explication task was to familiarize all of the participants with the model’s common cause, common effect, causal chain, and self-enhancing system. The participants received eight phenomena descriptions: four from the field of history, four from the field of electrical engineering. Each description was followed by the label of the underlying key causal model and a general explanation of this model. The explication of the example situation highlighted and explained how the elements in the example relate to the roles of the elements in the key causal model. In that sense, the explication structurally links a concrete example situation to the general functioning of the particular key causal model. In the original intervention, the participants were also asked to draw out the causal diagrams of the examples. We omitted the drawing of the causal diagram because the drawing was impossible to implement in the online experimental platform Labvanced. As Goldwater and Gentner [25] showed that the effect of the intervention was not diminished when simply providing participants with causal diagrams rather than asking them to draw them, we assumed this set-up would not affect the effectiveness of the intervention.

The structural alignment task involved four step-wise analogical comparisons. For each comparison, two analogs, i.e., two situations from different domains (electrical engineering and history), but with the same underlying causal structure, were presented. The participants were asked what the two situations had in common. They were provided with a table that listed the elements of one situation and were asked to assign the corresponding elements of the second situation, thus guiding them through the process of structural alignment.

**Tutorial**: The tutorial aimed at improving the conceptual understanding of the dynamic behavior of the key causal models as well as conveying procedural knowledge. This included information on how the system behaves as well as how it changes as a result of the manipulation of a variable. A thorough understanding of the implications of causal structures is crucial for the explanation and extrapolation of the system behavior [74]. It forms the basis for mental simulation [75], which is essential in order to derive suitable solution strategies during the *knowledge application* phase in the complex problem-solving task.

In the instructional design process of the tutorial, we systematically analyzed what specific factual knowledge would be necessary for controlling a system based on the key causal models. For example, in a common effect model, the value of the effect variable is affected by all other variables. Hence, the value of the effect variable is most efficiently increased by decreasing all cause variables with a diminishing causal relation and increasing all variables with an enhancing causal relation with the effect variable. Based on the identified relevant knowledge aspects, we designed ten multiple choice questions. We ensured that each aspect identified was captured at least once in the multiple-choice questions. Each multiple-choice question included three to four answer options. In each question, at least one answer option was correct and never all options.

**Figure 2:**
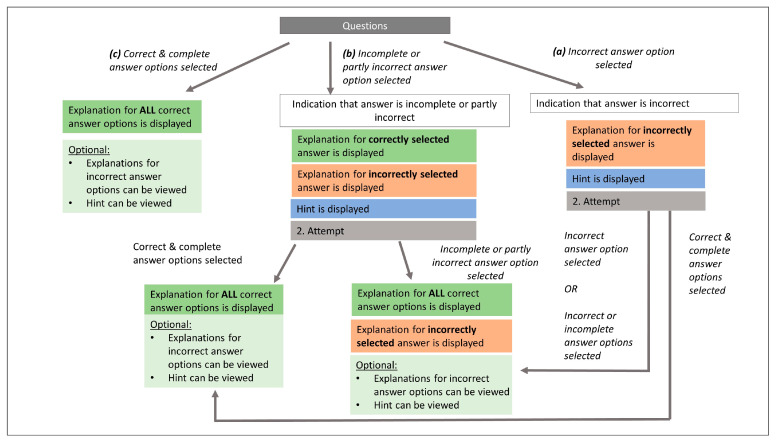
Informative Tutoring Feedback algorithm implemented into the tutorial questions.

We implemented Informative Tutoring Feedback (ITF) based on Narciss [76] into the tutorial tasks. The idea of ITF is to refrain from providing feedback in the form of knowledge of the correct result and rather to provide hints about possible sources of errors. This kind of elaborated feedback has been demonstrated to be more beneficial for learning than simply providing the correct solution [77]. Thus, we aimed to make the tutorial more effective with regard to learning than the inference question task in our previous study [27]. A feedback algorithm was implemented (see Figure 2).

(a) In case of an incorrect answer, the participants received feedback that their answer was incorrect and an explanation of why it was incorrect. Additionally, they were provided with a hint pointing to relevant aspects in the questions, thus helping participants to overcome deficient understanding or incorrect conceptions in order to help them correct their mistake on their own in a second attempt. After the second attempt, the participants were again informed about the correctness of their answer. This time, however, they also received corrective feedback in the form of the correct solution and explanations for each answer option.
(b) If the participant’s answer was either incomplete or partly wrong (i.e., at least one correct and one incorrect answer option was chosen), then they received feedback that their answer was incomplete or partly wrong. For all correct answer options, they were provided with an explanation of why it was correct, and for all incorrect answer options they received an explanation of why it was incorrect. The participants also received a hint on important aspects of the question to pay attention to. Then they could proceed with the second attempt of answering the question. After the second attempt, the participants were informed about the correctness of their answer. This time, however, they also received corrective feedback in the form of the correct solution and explanations for each answer option.
(c) In case the participants gave the correct answer, they received feedback that their answer was correct and also a short explanation as to why. Interested participants could access the explanations of all answer options as well as the hint. When finished reading, they could proceed to the next question.

**Figure 3:**
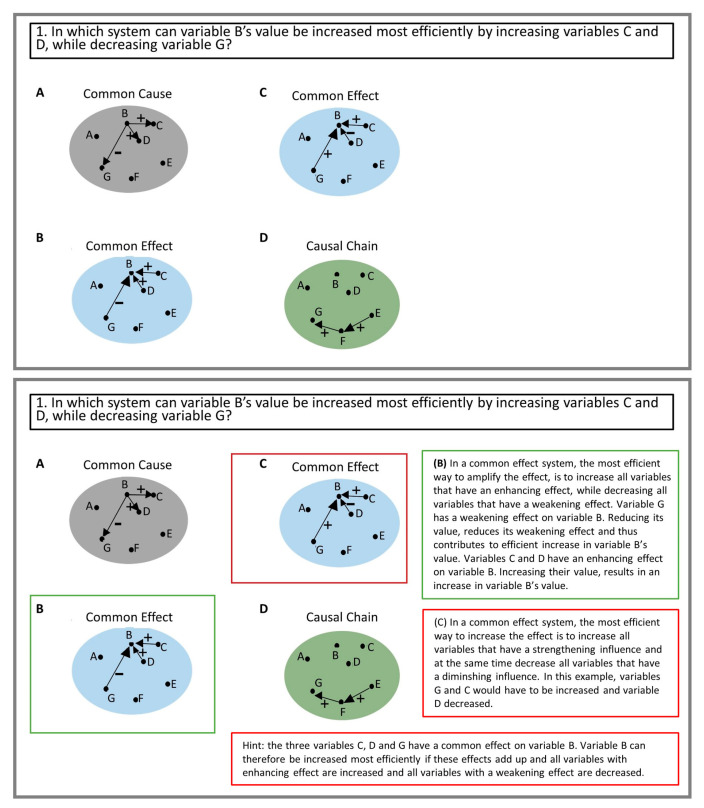
Above: Tutorial question 1. Below: Information provided after choosing one correct answer option (B) and one incorrect answer option (C).

Each option contained a depiction of a causal model (see Figure 3). In the intervention, we provided the participants with causal model diagrams for the example situations. We used concrete labels for the elements according to the specific instantiations in the situations. Goldstone and Son [78] demonstrated that transfer becomes more likely when introducing a concept using grounded representations of the agents (in their example, ants) and then switching to a more idealized representation (ants shown as dots). This is in line with the framework of Belenky and Schalk [79] who posit that grounded materials aid learning and immediate performance, whereas abstract materials are more difficult to learn in the first place, but are more beneficial for subsequent transfer. To account for this, we decided to use idealized representations of the key causal models in the tutorial questions.

We used letters as labels for the elements in the diagrams of the key causal models rather than concrete labels (as we did in the intervention). Further, it has been shown to be beneficial for transfer to introduce varied examples (for thematic variation, see D’Angelo & Trench [80]). Therefore, we provided the participants with varied causal models in terms of their spatial organization of elements and arrows as well as the number of variables involved (see Figure 3). We also provided participants with the corresponding key causal model labels for each causal diagram, because providing labels for the schema-governed categories has been argued to boost transfer [55,57] (see Figure 3).

The 11th question was a card sorting task that was designed as a summary of the content of the 10 multiple choice questions. The participants were presented with 13 cards, each containing a statement about one important aspect about the dynamic behavior of one of the key causal models. On the top of the page, the four causal model category labels were presented as column headers. The task was to sort each statement card to the matching key causal model by placing it under the according label. We included this task to boost the formation of schema-governed categories in terms of category status [55,81]. Different to the card sorting task employed by Kurtz and Honke [55], we predefined the categories and provided participants with the causal model category labels. We decided on that procedure, because a previous study using a category construction task as an instructional method yielded the best results for a subsequent transfer task if the category construction task was administered as a summary at the end of the lesson (as we did) and provided participants with the category labels (as we did) [82].

**Complex problem-solving task**: We used the MicroDYN approach as a measure for complex problem solving [67]. We decided to use MicroDYN for several reasons. First, it is a well-established measurement approach for complex problem solving with good psychometric properties [64,67]. MicroDYN is based on the multiple complex system approach that uses several microworlds with a minimalized time on task (3–5 min) as a composite measure for complex problem solving [67]. This set-up allows participants to be tested with several independent microworlds (i.e., items) that differ in their underlying causal structure, their semantical embedding, and complexity within one experimental session [67]. Multiple measurements improve the psychometric properties of the test because the measurement does not rely on one single data point [67]. The microworlds are based on structural equation models with input and output variables as introduced by Funke [61]. The number of variables and the causal relations between them can be defined by the researcher. They can be implemented between input and output variables, between output variables, and as self-enhancing effects on output variables, which adds a dynamic component irrespective of user intervention to the microworld [67]. The semantic embedding is created using imaginary denotations (fictitious plant or animal species) or arbitrary labels without deep meaning (e.g., Training A, B, C). Such domain-unspecific microworlds have the advantage of minimizing the influence of prior knowledge on task performance, and only the knowledge gathered during the exploration phase is mapped to performance [67].

These characteristics of the MicroDYN approach made it suitable for our study. It allowed us to create several microworlds based on the key causal models addressed in this study (as well as combinations thereof). The items are embedded in settings that make them independent of prior knowledge. We adapted ten items from the MicroDYN approach and changed the underlying causal structure to match them with the key causal models addressed in the intervention. Four of the items were based on one of the four key causal models each, and six were based on a combination of two key causal models each. This allowed the creation of a variety of complexity levels among the ten items, which is the core idea of the multiple complex system approach.

**Figure 4:**
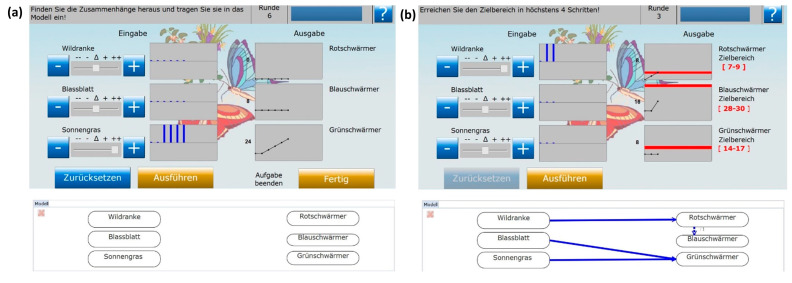
Screenshot of the microworld “butterflies” used in the experiment. Panel (**a**) shows the *knowledge acquisition* phase, panel (**b**) shows the *knowledge application* phase.

Scoring for the task was based on a dichotomous scoring for the two indicators *knowledge acquisition* and *knowledge application* for each item [67] (i.e., microworld, see Figure 4). Within each of the ten items, the participants received a score of one for the indication *knowledge acquisition* if the causal model that they drew during the exploration phase was correct (i.e., contained all correct relations and no spurious ones). In case a relation was missed or a spurious relation was erroneously added to the causal model during the *knowledge acquisition* phase, the participants were scored zero. For the indicator *knowledge application*, the participants were scored one if the goal states of all output variables were reached and zero if any of the output variables were outside of the goal state boundaries. The scores were directly computed and saved in the item log files. For analyses, the scores for the two indicators *knowledge acquisition* and *knowledge application* were averaged across the ten items per participant.

## 8. Results

### 8.1. Initial Causal Sorters

**Figure 5:**
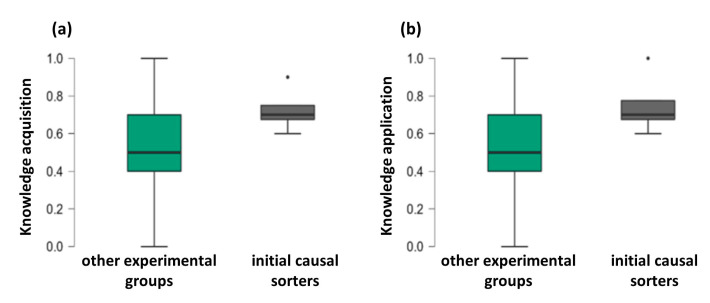
Comparison of performance of initial causal sorters and the rest of the sample with regard to (**a**) *knowledge acquisition* and (**b**) *knowledge application*.

The analysis of the initial ACST revealed that across all conditions, four (out of 183) participants sorted at least nine or more cards according to underlying key causal models and hence were classified as initial causal sorters. The percentage of initial causal sorters amounts to 2.2% in this sample, which is much lower than in the previous study with 11% [27]. Since we only identified four initial causal sorters in our sample, we refrained from conducting statistical analyses using this group. However, a descriptive inspection showed that the mean performance of the initial causal sorters (*knowledge acquisition M* = 0.73, *SD* = 0.13; *knowledge application M* = 0.75, *SD* = 0.17) was much higher than for the rest of the sample (*knowledge acquisition M* = 0.55, *SD* = 0.25; *knowledge application M* = 0.55, *SD* = 0.23, see Figure 5). The mean performance of the initial causal sorters was accordingly in the 70–80th percentile of the overall sample. This shows that the descriptive trend is in accordance with the assumption in hypothesis 1 that initial causal sorters are among the high performers in the sample.

### 8.2. Changes in the Ability to Detect Key Causal Models after Intervention and/or Tutorial

**Figure 6:**
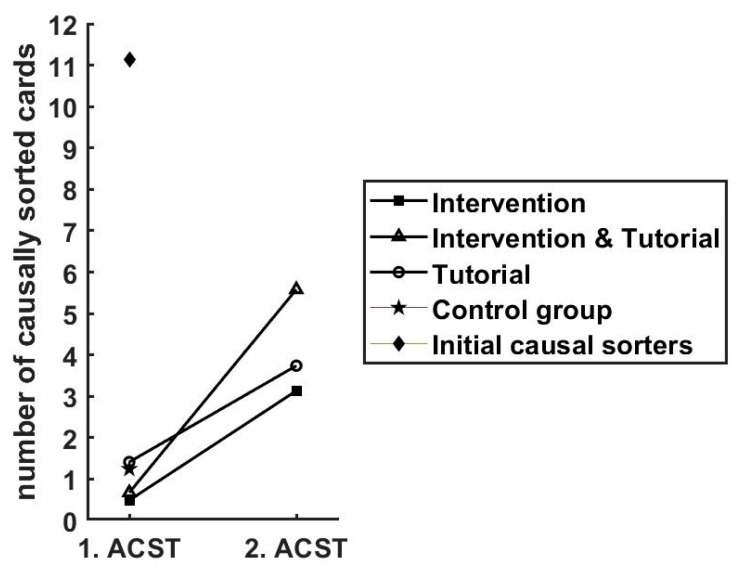
Frequencies of causal sorts in the Ambiguous Card Sorting Task (ACST) before and after the intervention, tutorial, or both. Note that the score of the ACST 1 includes data from the control group (CG; *n* = 45) and the initial causal sorters (*n* = 4). The slope for the treatment group that received both intervention and tutorial (Int and Tut) is steeper than for the groups that only received the intervention (Int) or the tutorial (Tut), indicating a stronger increase in the ability to recognize key causal models.

To test the effectiveness of the intervention and/or tutorial in enhancing the ability to detect key causal models, we analyzed the change in the number of causally sorted cards between the first and second ACST. The CG and the group of initial causal sorters only performed one ACST in order to limit any potential effects of the ACST with regard to building schema-governed category knowledge on these two baseline conditions. We conducted a mixed ANOVA with the two consecutive ACST measures as the within-subject factor and the treatment group (Int, Tut, Int and Tut) as the between-subject factor. The Levene’s test for equal variances revealed a violation of equality of variances (*p* < 0.05). As suggested by Hsu [83], we conducted post hoc tests as they are independent of this assumption. Using Holm-corrected post-tests, we found the number of causal sorts increased significantly in the second ACST compared to the first for each group (Int: MD = 2.64, *p* < 0.001; Tut: MD = 2.33, *p* < 0.001; Int and Tut: MD = 4.91; *p* < 0.001), i.e., confirming hypothesis 2a (see Figure 6). In order to determine whether the increase differed in dependence of the treatment group, we compared the differences between the scores in the second and the first ACST across the treatment groups (Int, Int and Tut and Tut). A Kruskal–Wallis Test revealed a significant main effect of group, *H*(2) = 10.21, *p* < 0.05. Dunn’s post hoc comparisons (Bonferroni-corrected) showed that participants who underwent both the intervention and tutorial (Int and Tut) had a significantly higher increase in the number of causal sorts in the ACST than the groups provided with only the intervention (*z* = 2.38, *p* < 0.05) or only the tutorial (*z* = 3.05, *p* < 0.05), i.e., confirming hypothesis 2b.

For completeness, we also determined whether the experimental groups differed with regard to the number of causally sorted cards in the first ACST (initial causal sorters were excluded as they should differ by definition). Because the assumption of normally distributed residuals was violated, we conducted a Kruskal–Wallis test on the score in the first ACST and compared it across groups. We found differences across groups, *H*(3) = 31.08, *p* < 0.001. Dunn’s post hoc comparisons (Bonferroni-corrected) indicated differences between group Int and Tut (*z* = −4.83, *p* < 0.001), Int and CG (*z* = −3.80, *p* < 0.001), Int and Tut and Tut (*z* = −3.80, *p* < 0.001), and Int and Tut and CG (*z* = −2.78, *p* < 0.05).

In order to show that the results in causal sorts are independent of the ACST versions administered (ACST and ACST_p), we conducted two additional analyses. First, we tested whether the number of causal sorts at the initial assessment time (i.e., first ACST before the treatments) differed between the two ACST versions (the order of the two versions was counterbalanced). The assumptions of normality and equal variances were violated; therefore, we conducted a Mann–Whitney *U* test. We found no differences in the number of causally sorted cards in the ACST (Mdn = 2.00) or the parallel version, ACST_p (Mdn = 1.50, *U* = 4140.00, *p* = 0.91, *r*_B_ = −0.01).

Further, we conducted a mixed ANOVA on the participants in the treatment groups with the order of the ACST versions (ACST first versus ACST_p first) as the independent variable and repeated measures on the number of causal sorts in the ACST (at assessment point one and two). While the increase in the number of causal sorts was significant, *F*(1, 132) = 89.21, *p* < 0.001, *η*^2^ = 0.21, this increase was independent of the order of the ACST versions administered, *F*(1, 132) = 0.35, *p* = 0.55, *η*^2^ < 0.001. These results indicate that there were no differences between the ACST versions in terms of the number of causally sorted cards and that the two ACST versions can therefore be considered parallel.

### 8.3. Complex Problem-Solving Performance across Experimental Groups

To determine the effect of the intervention and tutorial on the performance in the complex problem-solving task, we conducted a two-way MANOVA with the independent variables of intervention (yes/no) and tutorial (yes/no) on the performance indicators *knowledge acquisition* and *knowledge application*. We found no significant main effect for intervention, Pillai = 0.01, *F*(2,176) = 0.58, *p* = 0.56, for tutorial, Pillai = 0.02, *F*(2, 176) = 1.69, *p* = 0.19, or for the interaction, Pillai = 0.004, *F*(2, 176) = 0.32, *p* = 0.73. However, this result is reasonable because the earlier study [27] showed that the positive effects of the intervention were only observed in participants who were able to recognize the causal models reliably (i.e., causal sorters). The assumption is that the effect of the intervention and tutorial will only become visible in performance in *knowledge acquisition* and *knowledge application* if the participants show a substantial shift in causal sorting, i.e., classify as causal sorters after treatment. In the next step, we therefore determined the number of participants who shifted to a mainly causal sorting strategy and test the differences between causal and non-causal sorters in the treatment groups and compared their performance to the baseline groups CG and initial causal sorters.

In group Int and group Tut, seven participants were classified as causal sorters (Int = 7, 15.6%; Tut = 7, 15.2%), and in group Int and Tut, 16 participants (37.2%) were classified as causal sorters. A Chi-square test of independence was performed to examine the relationship between treatment and the number of causal sorters in the second ACST. The relationship was significant, *X*^2^(2, 134) = 8.01, *p* < 0.05, Cramer’s *V* = 0.24. The results indicated that more than twice as many participants in the group that received the intervention and tutorial shifted their sorting strategy to a mainly causal strategy compared to the groups who only received one of the treatments.

### 8.4. Complex Problem-Solving Performance of Causal Sorters versus Non-Causal Sorters in the Treatment Groups

**Figure 7:**
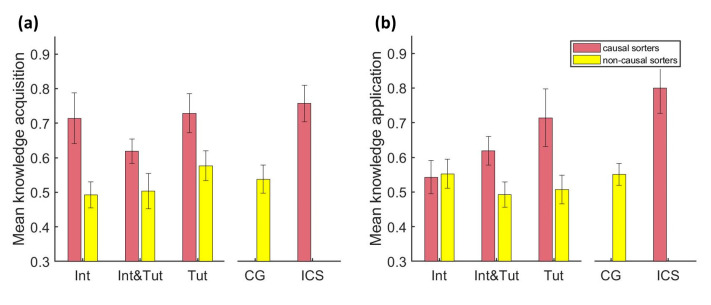
Complex problem-solving performance for causal and non-causal sorters according to experimental group for (**a**) *knowledge acquisition* phase and (**b**) *knowledge application* phase. Int = intervention, Tut = tutorial, CG = control group, ICS = initial causal sorters.

In order to determine whether shifting to a mainly causal sorting pattern after intervention and/or tutorial is associated with better performance in the complex problem-solving tasks, we conducted a two-way MANOVA with the factors of causal sorter (yes/no) and treatment condition (Int, Int and Tut, Tut) as the independent variables and *knowledge acquisition* and *knowledge application* as the dependent variables. The two-way MANOVA revealed a statistically significant multivariate main effect for the factor causal sorter, Pillai = 0.06, *F*(2, 127) = 4.00, *p* < 0.05, but no multivariate main effect for treatment condition, Pillai = 0.05, *F*(4, 256) = 1.62, *p* = 0.17, nor for the interaction, Pillai = 0.07, *F*(4, 256) = 2.31, *p* = 0.06. Follow-up ANOVAs revealed a significant main effect for the causal sorter factor for *knowledge acquisition*, *F*(1, 128) = 9.60, *p* < 0.01, *η*² = 0.07, but not for group, *F*(2, 128) = 1.11, *p* = 0.33, *η*^2^ = 0.02, and no interaction, *F*(2, 128) = 0.38, *p* = 0.69, *η*^2^ = 0.01 (see Figure 7).

This demonstrates that participants who shifted their sorting strategy to a predominantly causal strategy as a response to the treatment showed significantly better performance in the *knowledge acquisition* phase compared to those participants who did not shift their sorting strategy, confirming hypothesis 3a. The follow-up ANOVA for *knowledge application* revealed a similar pattern of results: a significant main effect for causal sorter factor, *F*(1, 128) = 4.56, *p* < 0.05, *η*^2^ = 0.03, no significant effects for treatment condition, *F*(2, 128) = 0.57, *p* = 0.57, *η*^2^ = 0.01 and no interaction, *F*(2, 128) = 1.37, *p* = 0.26, *η*^2^ = 0.02. Further, we also conducted a planned contrast between causal and non-causal sorters in group Int. The contrast yielded no significant result, *t*(128) = 0.10, *p* = 0.92., therefore showing that causal sorters in group Int did not perform better during *knowledge application* than non-causal sorters. Therefore, hypothesis 3b was confirmed that assumed that causal sorters who received the tutorial outperform non-causal sorters in *knowledge application*, while causal sorters who only received the intervention do not show better performance in *knowledge application* than non-causal sorters.

### 8.5. Complex Problem-Solving Performance of Causal Sorters versus Baseline Conditions CG and Initial Causal Sorters

In the next step, we wanted to relate the results to the two baseline conditions, i.e., the control group and initial causal sorters. If shifting to a mainly causal sorting pattern is beneficial, then the causal sorters in the three treatment groups should outperform the CG with regard to knowledge acquisition. In order to test this, we conducted a planned contrast between the combined performance of the causal sorters in the treatment groups and the CG. As equality of variance was violated, we used contrasts that do not assume equal variances. *Knowledge acquisition* was significantly higher for the (combined) causal sorters in the treatment groups compared to the CG (1 1 1 −3), *t*(52.12) = 2.85, *p* < 0.05, *d* = 1.91 confirming hypothesis 4a. A planned contrast on *knowledge application* on the combined performance of groups who received the tutorial (Int and Tut, Tut) against the CG (0 1 1 −2) revealed significantly better performance, *t*(71) = 2.14, *p* < 0.05, *d* = 1.16, supporting the assumption that the causal sorters who received the tutorial outperformed the CG in *knowledge application*, i.e., confirming hypothesis 4b. The contrast between the causal sorters of the group Int and the CG (1 0 0 −1) was not significant, *t*(71) = 0.10, *p* = 0.92, *d* = 0.04, supporting hypothesis 4c that assumed that causal sorters in the group that only receive the intervention do not perform better in *knowledge application* than the participants in CG.

We also wanted to test whether the performance of the causal sorters in the treatment groups was comparable to the performance of the initial causal sorters. Because the total number of initial causal sorters was rather small (*n* = 4), we refrained from conducting inference statistics and limited the analysis to descriptive observations. With regard to the *knowledge acquisition*, the causal sorters in the treatment groups showed comparably high performance as the initial causal sorters (Int: *M* = 0.71; Int and Tut: *M* = 0.62; Tut: *M* = 0.73; ICS: *M* = 0.73; see Figure 7, the red columns in panel a). Therefore, the descriptive trend is in accordance with the assumption in hypothesis 5a, which assumed no differences in *knowledge acquisition* between causal sorters in the treatment groups and initial causal sorters. For *knowledge application*, the causal sorters in the group that received the tutorial and intervention (Int and Tut) and tutorial (Tut) showed comparably high performance as the initial causal sorters, while the causal sorters in the group that only received the intervention (Int) showed lower performance (Int = 0.54; Tut = 0.71; Int and Tut = 0.62; ICS = 0.75; see Figure 7, the red columns in panel b). These results demonstrate that the descriptive trend is in accordance with the assumption in hypotheses 5b and 5c that assumed no differences between the causal sorters and initial causal sorters with regard to *knowledge application* in groups that received the tutorial (5b), but lower performance in the group that only received the intervention (5c).

### 8.6. Performance in the Card Sorting Task of the Tutorial

We also compared the performance in the card sorting task in the tutorial, i.e., the number of cards correctly assigned to the corresponding key causal model label between the two groups that received the tutorial. We conducted a Mann–Whitney U test that indicated no significant difference of correctly assigned cards in the group Int and Tut (Mdn = 10) and group Tut (Mdn = 10), *U* = 869.00, *p* = 0.32, *r*_B_ = −0.12. Therefore, hypothesis 6 that predicted a facilitation effect for learning in the tutorial content for the group that received the intervention prior to the tutorial was rejected.

We conducted an exploratory analysis to determine whether causal sorting in the ACST is associated with longer time on task. We correlated the time on task on the second ACST (for initial causal sorters and the CG the first) with the number of causally sorted cards and found a positive Spearman’s rank correlation *r* = 0.50, *p* < 0.001, indicating longer times on task with an increasing number of causally sorted cards.

## 9. Discussion

In this study, we investigated whether the expert-like ability to recognize key causal models across situations is associated with better performance in a complex problem-solving task. The guiding assumption was that the acquisition of the schema-governed category knowledge of key causal models facilitates the recognition of the according key causal models across situations as well as the activation of associated conceptual knowledge and should therefore facilitate problem solving in complex tasks based on the key causal models. To test this assumption, we applied a combination of learning tasks designed to foster the construction of schema-governed category knowledge and tested its effects on the ability to recognize key causal models and related it to complex problem-solving performance.

The key results of this study are as follows. First, we found that participants who spontaneously exhibited a high ability to detect key causal models (i.e., initial causal sorters) also showed high performance in the complex problem-solving subtasks *knowledge acquisition* and *knowledge application* and were, in fact, among the more successful problem solvers in the sample. This was an important result as it confirms the main idea of this experiment that organizing knowledge in terms of causal models is related to better complex problem solving in tasks that are based on these key causal models. It also substantiates the finding of our previous study [27] in which initial causal sorters were also among the top performers. In a next step, we investigated the differential effects of teaching schema-governed categories of the key causal models using the adapted invention by Goldwater and Gentner [25] and promoting deep conceptual understanding of the key causal models using the tutorial that we designed. Second, we replicated the effectiveness of the intervention in enhancing the ability to detect key causal models. Our results extended previous findings [25,27] as the two ACSTs included in the current study design allowed us to directly relate the increase in the ability to detect key causal models to the intervention (and/or tutorial). Third, we found no direct effect of the intervention and tutorial on performance in the complex problem-solving task. However, this is consistent with the expectation that performance gains will only be evident if participants are able to identify key causal models reliably after treatment. The participants who responded to the treatments in the intended way and shifted their sorting behavior to an expert-like predominantly causal sorting pattern were classified as causal sorters. The causal sorters outperformed non-causal sorters in the complex problem-solving task with one important exception: causal sorters in the group that only received the intervention did not show better performance during the *knowledge application* phase compared to non-causal sorters. This pattern of results demonstrates the incremental benefit of the tutorial in enhancing the conceptual understanding of the dynamic behavior of the key causal models and conveying the procedural knowledge necessary for successful controlling of the microworlds. It corroborates the assumption that the ability to reliably recognize key causal models will only be beneficial during *knowledge application* if participants also possess a deep conceptual understanding. In that case, they can use the categorization as a pointer to the relevant memory content linked to the key causal model labels as predicted by the category status hypothesis [55] and earlier accounts that have demonstrated that a recognition of the problem type can facilitate the activation and productive use of associated conceptual knowledge [24,60]. Fourth, we compared the performance of those participants who shifted to a predominantly causal sorting as a result of the treatments (i.e., causal sorters) to the two baseline conditions CG and initial causal sorters. In comparison to the CG, causal sorters showed better complex problem solving, except for the group that only received the intervention that did not show better performance in *knowledge application* (see point three for explanation). We found comparably high performance on a descriptive level, as the initial causal sorters (again, except for causal sorters in the group that only received the intervention that performed worse than initial causal sorters in *knowledge application*). This pattern of results suggests that it is possible (at least as an immediate effect) to induce an expert-like relational shift in participants with an easy and short intervention and tutorial and that this shift is associated with enhanced complex problem-solving performance. Fifth, we did not find any support for our hypothesis that undergoing the intervention prior to the tutorial facilitates learning of the tutorial content. However, it is conceivable that the card sorting task in the tutorial did not capture the amount of learning of the tutorial content reliably.

Taken together, the results of this study support the core idea that the acquisition of the schema-governed category knowledge of the key causal models enhances the ability to detect the according models across situations. Together with a deep conceptual understanding of the key causal models, this type of knowledge organization is associated with better complex problem-solving performance in the subtasks of *knowledge acquisition* and *knowledge application*. Our results complement previous studies showing that categorizing problems according to their underlying principle is associated with better problem solving [24,41,84] and that categorization based on relational content can be taught and practiced [25,59,60].

Nonetheless, our results also stress that simply teaching and practicing classification is not enough. In order to realize the full potential of categorizing according to the underlying relational content, appropriate conceptual knowledge and associated procedural knowledge must be available, which can then be activated in the course of categorizing. This became evident in our study, as the performance of causal sorters who only received the intervention was improved for *knowledge acquisition,* but not for *knowledge application*. These participants were enabled to detect the underlying causal models, but did not improve their conceptual understanding and did therefore not benefit from the classification for subsequent application of their knowledge. These results indicate that the building of expertise cannot be bypassed by practicing classification alone. In order to form rich category representations, learning tasks also need to encompass the practice of the concept itself and the development of according procedures in order to allow for its successful application. Similarly, Markman and Ross [85] argued that building a true and rich category representation requires not only classification tasks that foster distinguishing between categories, but also inference tasks that promote the understanding of the internal relations among category features. Nonetheless, the acquisition of abstract schemata and practicing to classify novel situations as instances of a particular schema-governed category seems to be a fruitful way to make knowledge more accessible and transfer more likely once a conceptual understanding is acquired [54,55,81]. Therefore, we suggest that the inclusion of classification exercises in educational programs would be a useful addition to traditional teaching if the goal is to be able to apply what is learned later in relevant situations [54].

The use of complex problem solving as the dependent variable for transfer in our study broadens the scope of applicability of the results. Oftentimes, static tasks are used as dependent variables in studies on transfer (but see [86]). Complex problem-solving tasks, however, encompass dynamic aspects and represents typical 21st century requirements [6].

It is conceivable that the positive effect of forming abstract schemas of the key causal models not only improves retrieval from memory, but also facilitates the process of relational thinking by reducing the load on working memory [87]. Gray and Holyoak [87] showed that alongside fluid intelligence measures, i.e., Ravens Progressive Matrices [88] the score in the Semantic Similarities Test (SST) [89] was a good predictor for relational reasoning. The SST is a measure of crystalized intelligence based on verbal relational knowledge [89]. On this basis, the authors argued that a broad knowledge base about semantic relations in long term memory can facilitate relational reasoning by relieving the load on working memory. Relational schemata essentially bind several elements that are causally linked to a relational chunk stored in the long-term memory [90]. This form of chunking reduces the amount of information that needs to be held in parallel in working memory during relational reasoning, thus reducing cognitive strain [91,92]. From that perspective, it is plausible that participants with lower working memory capacity and/or measures in fluid intelligence would particularly benefit from building abstract schemata while experiencing more difficulties in the construal of the representations in the first place [93]. Therefore, it is conceivable that the short learning tasks in our study did not provide sufficient support in building the schema-governed categories of the key causal models for all participants. In terms of future studies, it would be worthwhile to include inter-individual measures of, e.g., working memory capacity or fluid intelligence. This would allow for the investigation of whether participants with lower working memory capacity would benefit from more support during the schema abstraction process, e.g., in the form of scaffolding. Scaffolding could be implemented in the form of worked examples that demonstrate and support the structural alignment process between concrete analogous situations and/or between the abstract causal model and a concrete example situation. Similarly, Kubricht [30] demonstrated that participants with lower measures of fluid intelligence benefitted more from scaffolding in the form of animated source analogs with regard to the amount of spontaneous transfer.

### Limitations and Outlook

The learning tasks used in this study required the participants to deeply engage in the tasks in order to induce the intended effects. Thorough processing, however, is likely to result in longer times on task. It is possible that the participants who lacked motivation for the task did not engage deeply enough and, consequently, did not build up schema-governed categories or conceptual knowledge, which may account for less causal sorting in the ACST. Beyond that, we conjecture that the results of the ACST task itself are influenced by motivation. Sorting the situation descriptions according to their underlying causal models is likely to be more cognitively demanding than sorting by superficial similarity. We found that the amount of causal sorting is positively correlated with longer times on task. Therefore, it is possible that motivational issues account for the misclassification of “veiled” (initial) causal sorters who theoretically would have been able to sort by causal strategy in the ACST, but preferred to adopt a more time-saving, cognitively less demanding domain sorting strategy. Future studies could aim to mitigate the influence of intrinsic motivation by including some external incentives such as a monetary bonus for “good sorting solutions” (i.e., causal sorting). Another possibility would be to constrain the sorting in the ACST to a causal strategy by predefining the causal model categories. This way, the ACST results may better mirror the ability to detect the causal models with less confounding [41]. But at the same time, such a predefined sorting task may induce category construction processes and can therefore itself act as a kind of intervention [55], which may be problematic for the interpretation of the results.

While we concentrated on *knowledge acquisition* and *knowledge application* as indicators for complex problem-solving performance, the use of computer-simulated microworlds also included the recording of computer-generated log files that provide comprehensive process data on every interaction with the microworld. These data include information on which variables a participant changed, how they proceeded, number of exploration steps, number of control steps necessary to reach the goal, etc. [94]. The analyses of process data can be used to determine specific behaviors in the problem-solving process that underlie successful and unsuccessful problem solving in a much more detailed manner [94]. In previous studies, more successful performance in complex problem solving has been associated with the use of effective exploration strategies such as the VOTAT (vary-one-thing-at-a-time) strategy [94,95,96]. The log file data gathered in this study could be extracted and analyzed. It would be interesting to analyze how the strategic behavior in the problem-solving process relates to the ability to recognize key causal models. For example, analyzing the controller expenditure would allow the assessment of the efficiency of controlling the system during the *knowledge application* phase. Employing an efficient manipulation strategy would allow the participant to reach the goals in a minimum number of control steps, while a less efficient strategy would involve sub-optimal manipulations in the first control steps, which would require counteracting in the following steps. Thus, it would allow for a more fine-grained analysis of participants’ performance than solely relying on the *knowledge application* indicator alone.

The experimental design entailed that the intervention and/or tutorial groups were exposed to a higher work load because they had to perform more tasks. Therefore, complex problem-solving performance may be biased towards the CG and the group of initial causal sorters since they processed the complex problem-solving task directly after the first ACST. It may be worth including a control task for the CG that puts similar cognitive strain on participants as the intervention and tutorial. Another possibility would be to separate the intervention and the complex problem-solving tasks into discrete experimental sessions spread over the course of several days. Thus, it could also serve as a test for the temporal stability of the effects of the intervention and tutorial on the complex problem-solving performance.

Although the intervention and tutorial were effective in increasing the ability to recognize the key causal models overall, the efficiency in terms of the fraction of participants who changed their sorting behavior to a mainly causal sorting pattern was significantly lower than in the previous study. In Kessler et al. [27], 64% of the participants in the intervention group were classified as causal sorters, whereas only 16% were in this study (and 15% in Tut, 37% in Int and Tut). Theoretically, this could be due to the identification and separation of initial causal sorters at the beginning of the experiment. However, the percentage of initial causal sorters was also much lower than in the last study (2% versus 11% in the previous study) and, therefore, cannot account for the low percentage of causal sorters in the treatment groups. There are three main differences to the last study that may have contributed to the lower effectiveness of the intervention. First, this study was conducted as an online study, depriving the experimenter of the opportunity to supervise the processing of the materials. Second, the participants were presented with an initial ACST in this study in which most participants adopted a mainly domain sorting strategy. It is conceivable that the participants may have felt inclined to stick with their initial sorting strategy in the second ACST, rather than adapting it to a more causal strategy as a result of the treatments, possibly due to the costs associated with switching the strategy (e.g., compare to [97,98]). Third, in this study, the participants were provided with causal diagrams of the examples in the intervention, whereas in the previous study, they had to draw the causal diagrams themselves as part of the intervention. Future studies should investigate ways to make the intervention and the tutorial more effective for a larger proportion of participants. One possibility would be to include more tasks as part of an interleaved study program encompassing a longer time horizon. Previous studies have demonstrated the beneficial effects of distributed over massed practice on subsequent test performance [99]. This would also allow the investigation of how long the training effects last and whether it would be possible to reactivate prior knowledge using hints or repeating a learning task. Effectiveness may also be enhanced by prompting self-explanations as part of the learning tasks or as part of the ACST, e.g., by asking for an explanation of why a situation description belonged to a key causal model category [100,101,102].

Up to this point, we have found a positive association between the ability to detect the key causal models and complex problem-solving performance. In terms of future research, it would be useful to extend these findings by establishing a causal relationship, e.g., by the inclusion of a pre-test of complex problem-solving performance in order to test whether increases in causal sorting can account for increases in complex problem-solving performance. With the current experimental design, it cannot be ruled out that, in fact, another inter-individual difference factor such as cognitive capacity accounts for both the increase in causal sorting as well as high complex problem-solving performance. Specifically, the identification of participants as causal sorters might simply have been correlated with the identification of the most able participants. In this case, it would not have been the intervention and tutorial that affected the complex problem-solving performance, but inter-individual differences in cognitive capacity. In fact, measures of intelligence are positively correlated with performance in complex problem solving [103,104]. However, the combination of the findings that the initial causal sorters were among the better problem solvers, the effectiveness of the intervention and/or tutorial in improving the ability to recognize important causal models across different situations, and the better performance of the causal sorters all suggest that thinking in terms of key causal models is important and trainable. Overall, it would be useful to gain a clearer understanding of the cognitive underpinnings and implications of inter-individual differences in building schema-governed categories (of key causal models) and how it relates to, e.g., relational reasoning and fluid intelligence, motivation or prior knowledge. The results could inform the design of learning tasks tailored to learners’ characteristics [105,106].

In this study, we tested the effects of the intervention and tutorial on spontaneous transfer. It is possible that transfer could be improved if the goal of the intervention and tutorial were explained to the participants at the beginning of the experiment. This is consistent with accounts stressing the importance of the expansive framing of contexts for increasing the likelihood of transfer by encouraging students to think outside the predefined scope of use for a particular case [107,108]. By encouraging students to think about the applicability of the materials in other contexts or fields, at other times or places, the content is put into a larger context, thus highlighting the potential relevance for future problems, which, in turn, may make transfer more likely. Including instructions that ask participants to categorize novel situations in terms of underlying relational content and hence support the active use of the causal model categories may also promote the habitual interpretation of situations in terms of the causal model categories.

## 10. Conclusions

We found that participants who showed a high spontaneous ability to categorize novel situations in terms of their underlying key causal models (i.e., initial causal sorters) were among the better performers in a complex problem-solving task that involved these causal models. Further, our results suggest that it was possible to promote the ability to detect key causal models with relatively short learning tasks that fostered the formation of abstract schemata (i.e., intervention [25]) and the conceptual understanding of the causal models (i.e., tutorial). The participants who shifted their causal sorting to an expert-like level (i.e., causal sorters) as a result of the intervention and the tutorial outperformed the non-causal sorters in the subsequent complex problem-solving task in the subtasks *knowledge acquisition* and *knowledge application* (except for group Int, which is in line with the hypotheses). The causal sorters outperformed the CG and showed, on a descriptive level, comparable performance to the initial causal sorters. Again, the exception was for the subtask *knowledge application* for the group Int, who did not receive the tutorial and hence did not have the opportunity to elaborate their conceptual understanding of the key causal models. The findings highlight that being able to categorize novel situations according to their underlying key causal model alone is insufficient for enhancing the transfer of the according concept or relational content. For successful application, conceptual and procedural knowledge are also necessary to be included in the schema. Nevertheless, categorization training seems to be a promising way to promote transfer once learners have thoroughly understood the concept (in a domain) by paving the way to make the relevant knowledge structures more accessible. Tasks that help learners to organize their knowledge around the main principles will result in more effective problem solving. In terms of future studies, it would be useful to investigate how inter-individual differences and task design affect learning and the use of schema-governed category knowledge. The findings could inform the design of individualized learning tasks, thus helping to make knowledge transfer accessible to more people. By using a complex problem-solving task as the dependent variable for transfer, we extended the scope of the results to dynamic tasks that reflect some of the typical challenges of the 21st century.

## Data Availability

The data used to support the findings of this study are available from the corresponding author upon reasonable request.
